# Conscientious refusal or conscientious provision: We can't have both

**DOI:** 10.1111/bioe.13285

**Published:** 2024-03-22

**Authors:** Ryan Kulesa, Alberto Giubilini

**Affiliations:** ^1^ Department of Philosophy University of Missouri Columbia Missouri USA; ^2^ Oxford Uehiro Centre for Practical Ethics University of Oxford Oxford UK; ^3^ Wellcome Centre for Ethics and Humanities University of Oxford Oxford UK

**Keywords:** conscientious provision, conscientious refusal, professional obligations, symmetry thesis

## Abstract

Some authors argue that it is permissible for clinicians to *conscientiously provide* abortion services because clinicians are already allowed to *conscientiously refuse* to provide certain services. Call this the symmetry thesis. We argue that on either of the two main understandings of the aim of the medical profession—what we will call “pathocentric” and “interest‐centric” views—conscientious refusal and conscientious provision are mutually exclusive. On pathocentric views, refusing to provide a service that takes away from a patient's health is professionally justified because there are compelling reasons, based on professional standards, to refuse to provide that service (e.g., it does not heal, and it is contrary to the goals of medicine). However, providing that same service is not professionally justified when providing that service would be contrary to the goals of medicine. Likewise, the thesis turns out false on interest‐centric views. Refusing to provide a service is not professionally justified when that service helps the patient fulfill her autonomous preferences because there are compelling reasons, based on professional standards, to provide that service (e.g., it helps her achieve her autonomous preferences, and it would be contrary to the goals of medicine to deny her that service). However, refusing to provide that same service is not professionally justified when refusing to provide that service would be contrary to the goals of medicine. As a result, on either of the two most plausible views on the goals of medicine, the symmetry thesis turns out false.

## 1 INTRODUCTION

Conscientious provision for medical services that are illegal has recently been proposed to have a similar justification as medical refusals to provide services that are legal. Some authors argue that it is *professionally* permissible for clinicians to *conscientiously provide* abortion services, even if providing services would break the law, because clinicians are already allowed to *conscientiously refuse* to provide certain services to those who are legally eligible to receive them.^1^ Call this the *professional* symmetry thesis. Others have argued that, if conscientious refusal is legally protected, so should conscientious provision, at least for certain types of medical interventions despite the potential for internal contradictions in the relevant laws.^2^ Call this second claim, the *legal* symmetry thesis. For the purposes of this article, we largely set aside the *legal* symmetry thesis and limit our focus to providing reasons to reject the *professional* symmetry thesis.^3^ Hence, throughout the article, in the discussion on the symmetry thesis, we should understand that it is referring to the *professional* symmetry thesis.

We provide an argument, based on the goals of medicine, that the symmetry thesis is false.^4^ More specifically, we argue that on either of the two main understandings of the aim of medicine—what we shall call “pathocentric” and “interest‐centric” views—conscientious refusal and conscientious provision are mutually exclusive. The conclusion is relevant because both defenders and opponents of the right of conscientious objection in medicine risk being accused of inconsistency or hypocrisy when they defend their views without “biting the bullet.” Those who defend the ethical or professional permissibility of conscientious objection to abortion may not want to defend the conscientious provisions of abortions. Analogously, those who argue in favor of the ethical or professional permissibility of conscientious provision of abortion may not want to defend conscientious refusal to provide abortion. Our conclusion implies that there is no inconsistency or hypocrisy in either position. On the contrary, both sides adopt the most plausible option that is available to them.

It is important to point out that our conclusion says nothing about whether conscientious refusals and conscientious provision in medicine are ethically or professionally permissible, or the extent to which they should be legally protected. In fact, the two authors have opposite views on whether conscientious objection in medicine is professionally permissible and warrants legal protection and have argued for the respective positions elsewhere.^5^ The point here is simply that, contrary to what many have argued, endorsing the claim that it is professionally permissible to conscientiously refuse to provide certain services does not commit one to the position that conscientious provision is professionally permissible, and conversely, endorsing the claim that it is professionally permissible to conscientiously provide certain services does not commit one to the position that conscientious refusal is professionally permissible.

## 2 CLARIFYING THE SYMMETRY THESIS

There are a number of authors defending the symmetry thesis^6^ and comparatively few attacking it.^7^ The thesis has been articulated as follows: “if negative appeals to conscience in the case of abortion are justified, so are positive appeals.”^8^ Since these authors generally think “that whatever criteria justify protecting negative appeals to conscience regarding abortion also justify protecting positive appeals regarding abortion,”^9^ we assume that the thesis is best expressed as a biconditional: conscientiously refusing to provide a service is justified if and only if, and to the same extent as, conscientiously providing that same service is justified. The symmetry thesis, however, is ambiguous between *moral* justification and *professional* justification. We consider each possible disambiguation in turn.

The symmetry thesis understood in terms of *moral* justification yields the following disambiguation: “conscientiously refusing to provide some service is *morally* justified if and only if, and to the same extent as, conscientiously providing that same service is *morally* justified.” However, once we fix the moral permissibility of the service itself, there are counterexamples to this disambiguation of the symmetry thesis.^10^ Suppose that, say, third‐trimester abortions are *not* morally permissible. If we assume this, then one would be morally justified in conscientiously refusing to provide that service, but it would not follow that she would be morally justified in conscientiously providing that service. This seems to be the case even if there was some prima facie moral obligation to always follow one's conscience. Surely any moral obligation to follow one's conscience is, other things being equal, outweighed by a moral obligation not to kill an innocent individual with a right to life.

If we instead assume that third‐trimester abortions are morally permissible, then it would be morally justifiable to provide third‐trimester abortions. However, it would not follow that one would be morally justified in conscientiously refusing to provide third‐trimester abortions. The morality of terminating the life of a fetus is not the only consideration at stake. Women's bodily autonomy is itself a moral principle that carries some weight. One might think that the wrongness of terminating the life of a fetus morally outweighs a woman's bodily autonomy, but if, ex hypothesi, third‐trimester abortion is not wrong, then refusing to provide abortion would infringe upon a rather uncontroversial moral principle for no good enough reason. Conscientiously refusing to provide third‐trimester abortions would not be morally justified, even if conscientiously providing it is morally justified. Thus, the moral version of the symmetry thesis runs into a significant problem once one fixes the moral permissibility of the service itself.

As a result, we assume that the thesis more plausibly refers to a provider's refusal or provision being *professionally* justified. A course of action is professionally justified if there are compelling reasons, based on the goal(s) of the profession, to pursue that course of action. A course of action can be either an action, a series of actions, inaction, or a combination of these. The extent to which a reason, based on the goals of the profession, is “compelling” will depend on various factors, such as whether or not there are countervailing considerations also based on the goals of the profession that override the reasons given to provide some service. For the purpose of this article, we don't need to give a precise account of what reasons, based on the goals of the profession, are compelling and which ones are not. All we need are clear examples of a professional course of action that is justified primarily by an appeal to the goals of that profession. Suppose, for instance, that it is a goal of the law profession to determine whether or not, or to what extent, one has broken the law, and what a fair sanction would be. If this is right, then a defense lawyer would be professionally justified in requesting that his defendant's court appearance be postponed until all the relevant evidence sufficient to provide a fair defense for his client has been collected. There are clear examples in medicine as well. Suppose that the goal of medicine is to *heal* people. If this is true, then clinicians might (implicitly or explicitly) appeal to this goal as a reason to provide vaccines, routine check‐ups, chemotherapy, palliative care, and vast array of services. We assume that an appeal to this goal, in the absence of countervailing considerations, is a very compelling reason to provide such services.

There may be cases, however, where the reasons (based on the goals of the profession) to pursue some course of action are not compelling. Consider a case in the law profession. Suppose that the relevant evidence for a case has largely been collected, and the defense is merely attempting to delay the proceedings. It seems that requesting a postponed trial date is not supported by a compelling enough reason, at least not one based on the goal(s) of the profession, one of which is to provide a fair trial. As will become clear at the end of our discussion, these sorts of cases will not affect our main claim, namely, that the professional symmetry thesis is false. If, therefore, some readers do not find the examples we provide as having provided compelling reasons to act in some way (based on the goals of the profession), they can simply substitute it for a case in which it is clear, to them, that there are compelling reasons (based on the goals of the profession) to act in that way. Here we simply assume that having compelling reasons, based on the goal(s) of the profession, to pursue that course of action is sufficient for that course of action to be professionally justified.

Yet, this sufficient condition for professional justification does not necessarily tell one when a course of action is *not* professionally justified. Here, we follow Hershenov^11^ and assume that a course of action is *not* professionally justified if that course of action is *contrary to* the goals of that profession. One's (in)actions are contrary to the goals of the profession if, by that (in)action, one intends to hinder another's ability to achieve those goals through the services offered by the profession. Take, again, the law profession. If out of spite for a person on trial, a judge did not allow clearly relevant evidence to be presented to the jury during a trial, that judge would be acting *contrary to* the goals of the law profession by hindering the defendant's ability to participate in a fair trial.

Given this clarification on how we understand professional justification, we can clarify the symmetry thesis a bit more:

*Professional symmetry thesis*: Conscientiously refusing to provide some service is *professionally* justified if and only if, and to the same extent as, conscientiously providing that same service is *professionally* justified.

## 3 TWO VIEWS ON THE GOALS OF MEDICINE

In this section and the next, we argue that, given either of two plausible views of the appropriate goals of medicine and of health care, conscientious refusal and conscientious provision are mutually exclusive and that, therefore, the Professional Symmetry Thesis is false given either view.

We are assuming that healthcare professionals ought professionally to do what falls within the appropriate scope of medicine and health care, and they ought not to do what is contrary to the goals of medicine. The big question is, of course, what such an appropriate scope consists of. On some services, professionals disagree. Those who conscientiously refuse to provide certain services may do so because they conscientiously believe those services are *contrary* to the appropriate goals of medicine and health care and therefore there is no professional obligation to provide them, even if those services are legally available and endorsed by their professional organizations.^12^ Likewise, those who conscientiously provide certain services may do so because they believe those services do fall within the appropriate scope of medicine and health care and therefore there is a professional obligation to provide them, even if those services are not legally available.

However, conscientious refusals are protected, normally, when there is deemed to be reasonable disagreement over a certain practice which touches upon certain moral views, particularly those concerning the value and meaning of life and death. As mentioned, equal protection is not offered to conscientious provision. Those defending the symmetry thesis have typically relied on the value of conscience and the importance of freedom of conscience to sustain the equivalence of positive and negative claims: if *conscience* (and conscience alone) warrants ethical, professional, or legal profession, it does so whether it manifests itself as conscientious refusals or conscientious provisions, so they argue.^13^

However, a principle of freedom of conscience by itself is not sufficient, and perhaps not even necessary, to ground the professional permissibility of conscientious refusal. If it was sufficient, health care professionals would be professionally justified in refusing to provide any service whatsoever as long as they have some conscientious objection to them—including, for example, vaccination, painkillers, antibiotics, blood transfusions, and so on. Not many defenders of conscientious refusals would subscribe to this view, as it seems to imply a *reductio* of their position. Typically, those who defend a right to conscientious refusal apply their arguments to specific procedures that are, at the very least, consistent with some plausible view of professional obligations, the moral (im)permissibility of which is subject to reasonable disagreement. For instance, most of the discussion surrounding conscientious refusals centers around a clinician's ability to refuse to provide morally contentious services such as abortion,^14^ end‐of‐life decisions,^15^ emergency contraception,^16^ and various treatments for gender dysphoria.^17^ In this sense, their arguments and conclusion match the legal frameworks that currently regulate conscientious refusals, as presented above. Freedom of conscience might play a role in these arguments, and perhaps it is a necessary principle to justify the professional permissibility of *conscientious* refusal. But it cannot be sufficient. If a principle of freedom of conscience is not sufficient to ground the professional permissibility of refusals, an alternative to or supplement of such a principle will likely need to appeal to what it is that grounds one's professional obligations. Of course, such refusals may still be properly referred to as *conscientious* refusals since clinicians' consciences often play a role in their decision to refuse to provide a service. The only claim we're making is that such refusals cannot be *professionally* justified based *solely* on the clinician's conscientious convictions.

We think the most plausible views of what grounds one's professional obligations make an explicit appeal to the appropriate goals of medicine and of health care. This point has been recognized by both the opponents^18^ and defenders^19^ of conscientious refusals. If and when conscientious refusal is professionally justified, it is not only because clinicians are following their conscience but also because the services clinicians refuse to provide are not considered professionally obligatory.

What, then, are the goals of medicine? There are a variety of nuanced views, but, for the purpose of this discussion, we will focus on two families of views which we will refer to as “pathocentric” and “interest‐centric” views. The use of these broad categories should not be taken to suggest that the different views that can be grouped under each heading do not differ in important ways—for example, on interest‐centric views, whether or not a service is in the patient's “best interest” will probably vary depending on one's preferred theory of well‐being. We are only claiming that these differences are not relevant for the purpose of defining the legitimate scope (if any) of conscientious refusals and provisions in medicine.

According to pathocentric views of medicine and health care, the goal of medicine and of health care is to *heal patients*, where “healing” is understood in a broad sense to include treating or preventing pathological conditions, reducing their severity, or mitigating their bad effects.^20^ These accounts usually presuppose that the malfunction of an organism's parts or processes is both *necessary* and *sufficient* for one to have a pathology.^21^

On these views, an individual's part or processes malfunctions just in case it makes a suboptimal contribution to survival and reproduction. “Suboptimal” could be defined against different standards of normality. For instance, in terms of species‐specific statistically normal functioning.^22^ Or in teleological terms, typically grounded on natural law theory or Aristotelian views, where different organs and body parts fulfill specific functions.^23^ Examples of services that achieve the goals of medicine on this view include vaccines, routine check‐ups, chemotherapy, and palliative care, but do not include things like abortions of healthy pregnancies or medical assistance in dying.^24^

On the other hand, what we will refer to as *interest‐centric* views of the goal of medicine claim that the goal of medicine is to help patients achieve what is in (some understanding of) their *best interests*, where something is in the patient's “best interest” if it helps increase the patient's well‐being.^25^ Given that there are multiple theories of well‐being on offer, interest‐centric accounts might be filled in with different views of well‐being, including those which base it on preference satisfaction and on patient autonomy.

The scope of well‐being that falls within the purview of the medical profession, we assume, cannot be restricted to healing understood as restoring or maintaining physiological and/or psychological well‐functioning, since the proposal would then collapse into a pathocentric view. However, if these views claim that the goal of medicine is to increase one's well‐being *full stop*, then the scope of medicine is far too broad. It would include, for instance, helping a homeless person find housing or telling a patient how to complete her tax returns. Now, one might say that while that is not part of the goals of medicine, it might still be part of the scope of health care (which is broader than medicine), for instance, because housing has an impact on people's health. While it is true that sometimes hospitals employ social workers to assist homeless people with finding shelter,^26^ that is a matter of social care more broadly understood—in fact, social workers are employed for that purpose, rather than healthcare professionals, which suggests that the task fall outside the proper scope of healthcare specifically, and therefore *of health*care professionals' responsibilities. To keep the scope from ballooning in this way, we assume that interest‐centric views limit the scope of medicine and health care to increasing the patient's well‐being *through the use of* one's knowledge of the body and/or of the use of tools that affect one's physiological condition. While a defense of this more limited view is beyond the scope of this article, we assume that this restriction, or something like it, is the most plausible interpretation of interest‐centric views. Thus, on an interest‐centric view, not only is it within the scope of medicine to provide services that address pathological conditions, such as vaccines, routine check‐ups, chemotherapy, and palliative care, but also those that may not, such as abortions, medical assistance in dying, and others.

## 4 CONSCIENTIOUS REFUSAL OR CONSCIENTIOUS PROVISION: WE CAN'T HAVE BOTH

Recall, now, the symmetry thesis:

*Professional symmetry thesis*: Conscientiously refusing to provide some service is professionally justified if and only if, and to the same extent as, conscientiously providing that same service is professionally justified.

If either the pathocentric view or interest‐centric view is correct, then the symmetry thesis is false.

### 4.1 The symmetry thesis is false on a pathocentric view of medicine

To see why, suppose that the goal of medicine is pathocentric, that is, the goal of medicine is to treat or prevent, reduce the severity of, or mitigate the bad effects of pathologies, defined in terms of some parameter for normal functioning. If one supports this view, then some conscientious refusals can be professionally justified. Recall that a course of action is professionally justified if there are compelling reasons, based on the goal(s) of the profession, to pursue that course of action. Consider abortions in the case of healthy pregnancies. Refusing to provide abortions is professionally justified, according to the pathocentric model, because these services do not help the patient achieve the goal of medicine, which is health. The fact that abortion does not help achieve, and is *contrary to*, the goals of medicine is a compelling reason, on the pathocentric view, not to provide the service. Pregnancy is indeed a sign of good health: the capacity to become pregnant is statistically normal in women within a certain age range and, on either a biostatistical or teleological view of health, pregnancy fulfills the function of a woman's reproductive organs. As a result, conscientious refusals are professionally justified—indeed professionally required—on a pathocentric view of the goals of medicine and health care.

As we noted above, part of the explanation of why, on a pathocentric view, there is a compelling reason (based on the goal of the profession) to *not* provide abortions is that abortions are *contrary to* the goal of medicine. To provide these services would be to act *contrary to* the goals of medicine because they hinder a patient's ability to achieve the goals of medicine through the services it offers. These detract from a patient's health by reducing at least one of her parts or processes below typical functional ability or by suppressing some of their functions.^27^ To be clear, this view *does not* entail that there is *no* reasons that can justify providing services that are contrary to the goals of medicine, as defined by the pathocentric model, such as abortion. Rather, the view is that such reasons cannot be based on professional obligations as defined by the appropriate goals of medicine and health care. One may well be ethically justified, all things considered, in providing services that detract from a patient's health as defined by the pathocentric model. Our point is that the justification is not professional in nature, because professional obligations are defined by the appropriate goals of medicine and health care which, on the pathocentric model, do not include those services.

Indeed, on the pathocentric model, conscientious refusal to provide certain services is not only permissible, but professionally required, given that to the extent that they disrupt normal functions of the organism, they introduce pathological states. If that is true, it follows that conscientious provision of the very same services is not professionally justified because the provision of such services contravenes the proper goals of medicine and health care. What we have just said certainly applies to refusals and provisions of services which detract from the organism's all‐things‐considered proper functioning, like abortions of healthy pregnancies, medical assistance in dying, vasectomy, and so on.^28^ For these procedures, we can confidently say that, on the pathocentric model, conscientious refusals to provide many services are professionally justified whereas the conscientious provision of those same services is *not* professionally justified.

### 4.2 The symmetry thesis is false on an interest‐centric view of medicine

The symmetry thesis also turns out false, given interest‐centric views of medicine. Interest‐centric views claim that the primary goal of medicine is to increase patient well‐being through the use of one's knowledge of the body and/or the use of tools that affect one's physiological condition. On a version of it, the goal of medicine and health care is to fulfill the autonomous preference patients express for a certain service, at least as long as the patient is legally eligible to obtain it and there are no competing professional standards that prevent fulfilling those preferences, such as fair allocation of scarce resources. If an interest‐centric view is correct, then conscientious provision can be professionally justified, and indeed professionally required, whenever the service promotes the patient's best interest, as defined above.

Yet, in the same situations, conscientious refusals are *not* professionally justified given an interest‐centric view of medicine. Refusing to provide medical services that increase patient well‐being would be to act *contrary to* the goals of medicine and health care because it would hinder one's ability to achieve the goals of medicine through the services it offers. For instance, refusing to provide a woman an abortion to terminate a healthy pregnancy would be to act contrary to the goals of medicine, on interest‐centric views, because it would stop her from fulfilling her autonomous preferences. Of course, this example assumes a preference satisfaction interpretation of the interest‐centric accounts on offer, but the defender of a different version of an interest‐centric account can supply a different example depending on her preferred theory of well‐being. For example, if autonomy is an element of an objective‐list view of well‐being, then a women's autonomous decision to have an abortion may be in her best interest (assuming she does not have a greater interest in other goods on the list, e.g., physiological and psychological well‐functioning), and therefore generate a professional obligation on a doctor to provide it.

So, if an interest‐centric view of medicine is correct, conscientious refusals to provide many services are *not* professionally justified whereas the conscientious provision of those same services is professionally justified. Therefore, the symmetry thesis is false.

It seems that no matter which view of the goals of medicine you adopt, conscientious refusal and conscientious provision are mutually exclusive.

## 5 CONCLUSION

We have argued that a view that is often implied and sometimes explicitly defended in discussions on conscientious objection in medicine is false. That is what we have called the professional symmetry thesis. According to this view, for any medical service that a healthcare professional is qualified to provide, those who think conscientious refusal is professionally justified (or even required) also need to commit to the view that conscientious provision of the same service is professionally justified. And conversely, those who think that it is professionally justified (or even required) to conscientiously provide a certain service need to commit to the view that is also professionally justified to refuse to provide it. In both cases, a principle of freedom of conscience seems to provide equally strong justification for the objection in the form of refusal and of provision.

However, we have argued that neither side of the debate needs to commit to this view. That is because a principle of freedom of conscience in support of conscientious objection—either as refusal or as provision—needs to be supplemented (or perhaps replaced) by reference to a professional obligation to provide all (and on some views, all and only) the services that fall within the proper scope of the profession. As we have argued, this relatively uncontested professional obligation implies that the symmetry thesis is false.

Indeed, not only does a position in support of conscientious refusal not imply support for conscientious provision, and vice versa, but it also seems that the two positions are mutually exclusive (at least, given either a pathocentric or interest‐centric view of medicine): if conscientious refusal in medicine is professionally permissible in certain cases, then conscientious provision is not professionally permissible in those same cases; and if conscientious provision in medicine is professionally permissible in certain cases, then conscientious refusal is not professionally permissible in those same cases.

There are other ways to refute the symmetry thesis, but these have been cashed out in terms of claim rights or in terms of possible legal frameworks. Abram Brummett,^29^ for instance, argues that the symmetry does not hold because a right to conscientious refusal is a negative right, while a right to conscientious provision would be a positive right. Quite simply, conscientious refusals would not require institutions and organizations to do anything to be accommodated, while conscientious provision would require institutions and organizations to take active steps to guarantee that the conscientious provider can perform an otherwise prohibited procedure. Since negative rights are usually stronger than positive rights, conscientious refusals and provisions are not morally equivalent. Some have questioned this framing.^30^ In any case, Brummet's justification is based on the moral weight of different types of claims of conscience and can be challenged or defended on those separate grounds.

While, as Brummet demonstrates, there are other ways to deny the symmetry thesis, we are sure there may be other ways to defend it as well. For instance, one might attempt to save the symmetry thesis by adopting a different account of the goals of medicine. We leave this possibility a live option. Perhaps a constructivist view of medicine will allow for the professional permissibility of both conscientious refusals and provisions.^31^ We have made a more limited point: on either of (what we think are) the two most plausible views of the goals of medicine, conscientious provision and conscientious refusal are mutually exclusive.

## CONFLICT OF INTEREST STATEMENT

The authors declare no conflict of interest.

## Footnotes

1 Harris, L. (2012). Recognizing conscience in abortion provision. *New England Journal of Medicine*, *367*(11), 981–983; Fritz, K. (2021). Unjustified asymmetry: Positive claims of conscience and heartbeat bills. *The American Journal of Bioethics*, *21*(8), 46–59; Ryan, I., Premkumar, A., & Watson, K. (2022). Why the post‐*Roe* era requires protecting conscientious provision as we protect conscientious refusal in health care. *AMA Journal of Ethics*, *24*(9), E906–E912.

2 Fox, D. (2023). Medical disobedience. *Harvard Law Review*, *136*(4), 1030–1111; Wicclair, M. (2009). Negative and positive claims of conscience. *Cambridge Quarterly of Healthcare Ethics*, *18*(1), 14–22.

3 Both Wicclair (ibid) and Fox (ibid) appeal to the value normally conferred to freedom of conscience in legislation and argue that, for the sake of consistency, if that principle justifies legal protection of conscientious refusal, it also justifies legal protection of conscientious commitment. This view falls prey to an obvious problem—which neither author addresses in detail—that a law that prohibits a practice while allowing its conscientious provision would contravene the point of the law (whatever its merits). There might be ways to address this concern—for example, by considering consistency with professional standards a mitigating circumstance when conscientious provision breaks the law. This would deserve a separate discussion. Whatever the merits of these two proposals, they would need to address objections on moral, legal, or conceptual grounds which our argument can avoid. Our argument is merely about the professional permissibility of conscientious refusal and provision and is based on considerations around professional obligations and the appropriate scope of medicine.

4 We use “the goal of medicine” as shorthand for “the goal of the medical profession.”

5 Giubilini, A. (2017). Objection to conscience: An argument against conscience exemptions in healthcare. Bioethics, 31, 400–408. https://doi.org/10.1111/bioe.12333; Kulesa, R. (2022). A defense of conscientious objection: Why health is integral to the permissibility of medical refusals. Bioethics, 36, 54–62. https://doi.org/10.1111/bioe.12956

6 Harris, op. cit. note 1; Fritz, op. cit. note 1; Ryan et. al., op. cit. note 1; Fox, op. cit. note 2; Wicclair, op. cit. note 2; Buchbinder, M., Lassiter, D., Mercier, R., Bryant, A., Lyerly, A. (2016). Reframing conscientious care: Providing abortion care when law and conscience collide. *Hastings Center Report*, *46*(2), 22–30.

7 Brummett, A. (2020). Should positive claims of conscience receive the same protection as negative claims of conscience? Clarifying the asymmetry debate. *The Journal of Clinical Ethics*, *31*(2), 136–142.

8 Fritz, op. cit. note 1, p. 50.

9 Ibid: 47.

10 Here, we assume that moral relativism is false. If one endorses some form of moral relativism, then, ceteris paribus, both the claim that abortion is morally impermissible and the claim that abortion is morally permissible are true. The former claim would morally justify (and indeed require) conscientious refusal and the latter would morally justify conscientious provision (and indeed require it if there are other reasons in favor of it). We are not excluding the possibility that this form of relativism is true, but even if it is, it makes the moral version of the symmetry thesis problematic: it would always be permissible to refuse or to provide services as long as one conscientiously believes that that is the right thing to do. This seems to be a reductio ad absurdum as it would imply that literally any provision or any refusal to provide any service is morally permissible.

11 Hershenov, D. (2021). Conscientious objection or an internal morality of medicine? *Christian Bioethics: Non‐Ecumenical Studies in Medical Morality*, *27*(1), 104–121; Hershenov, D. (2020). Pathocentric health care and a minimal internal morality of medicine. *The Journal of Medicine and Philosophy: A Forum for Bioethics and Philosophy of Medicine*, *45*(1), 16–27.

12 Curlin, F., & Tollefsen, C. (2021). *The way of medicine: Ethics and the healing profession*. University of Notre Dame Press.

13 Harris, op. cit. note 1; Fox, op. cit. note 2; Wicclair, op. cit. note 2.

14 Minerva, F. (2015). Conscientious objection in Italy. *Journal of Medical Ethics*, *41*(2), 170–173.

15 McDougall, R., White, B., Ko, D., Keogh, L., & Willmott, L. (2022). Junior doctors and conscientious objection to voluntary assisted dying: ethical complexity in practice. *Journal of Medical Ethics*, *48*(8), 517–521.

16 Card, R. (2007). Conscientious objection and emergency contraception. *The American Journal of Bioethics*, *7*(6), 8–14.

17 Kulesa, R. (2023). Toward a standard of medical care: Why medical professionals can refuse to prescribe puberty blockers. *The New Bioethics*, *29*(2), 139–155.

18 Savulescu, J., & Schuklenk, U. (2017). Doctors have no right to refuse medical assistance in dying, abortion or contraception: doctors have no right to refuse medical assistance in dying, abortion or contraception. *Bioethics*, *31*(3), 162–70; Schuklenk, U., & Smalling, R. (2017). Why medical professionals have no moral claim to conscientious objection accommodation in liberal democracies. *Journal of Medical Ethics*, *43*(4), 234–240.

19 Hershenov, op. cit. note 10; Kim, E., & Ferguson, K. (2022). Conscientious objections, the nature of medicine, and the need for reformability. *Bioethics*, *36*(1), 63–70.

20 Hershenov, op. cit. note 10; Curlin and Tollefsen, op. cit. note 11; Kulesa, op. cit. note 16; Kulesa, op. cit. note 5; McAndrew, S. (2019). Internal morality of medicine and physician autonomy. *Journal of Medical Ethics*, *45*(3), 198–203.

21 Boorse, C. (1977). Health as a theoretical concept. *Philosophy of Science*, *44*(4), 542–573. But the position need not take on such a view of disease. For instance, if the concepts of “health” and “disease” are (at least party) socially constructed, then the pathocentric view is best put in terms of physiological and psychological (i.e., biological) malfunction. A defender of the pathocentric view can claim that the goal of medicine is to prevent biological malfunction, reduce the severity of such malfunction, or mitigate its bad effects. In this way, the pathocentric view can adopt either a biostatisical Boorse or a harmful dysfunction account of health and disease such as that proposed by Wakefield, J. (1992). Disorder as harmful dysfunction: a conceptual critique of DSM‐III‐R's definition of mental disorder. *Psychological Review*, *99*(2), 232–247 to the same effect. For instance, if a defender of the pathocentric view of medical refusals adopts Boorse's biostatistical definition of disease, then there is a compelling reason, given the goals of medicine, to refuse to provide an abortion since doing so would be contrary to the nature of medicine (i.e., it induces a pathology). Or, if the defender of the pathocentric view adopts a harmful dysfunction account of disease, then there is a compelling reason, given the goals of medicine to refuse to provide since doing so would be contrary to the nature of medicine (i.e., it induces a biological dysfunction), and this is true even if abortions are not, strictly speaking, pathology inducing (say, because they are not harmful dysfunctions).

22 Ibid.

23 Curlin and Tollefsen, op. cit. note 11; Kass, L. (1975). Regarding the end of medicine and the pursuit of health. *Public Interest*. *40*, 11–42.

24 We include palliative care in this list because services that *mitigate the bad effects of pathologies* have been included as pathocentric services in the standard characterization of the view as presented by its opponents (e.g., Boorse, C. (2016). Goals of medicine. In Élodie Giroux, *Naturalism in the Philosophy of Health* (pp. 145‐177). Springer International Publishing) as well as its defenders (e.g., Hershenov, *op. cit.* note 10; Kulesa, *op. cit.* note 19).

25 Boorse, op. cit. note 23; Giubilini, A., Schuklenk, U., Minerva, F., Savulescu, J. (2023). Conscientious commitment, professional obligations and abortion provision after the reversal of *Roe v Wade*. *Journal of Medical Ethics*, Epub ahead of print. https://doi.org/10.1136/jme-2022-108731

26 We thank an anonymous reviewer for this observation.

27 Hershenov, x. note 10.

28 Some services provided to alleviate gender dysphoria may or may not be included in this list. For instance, puberty blockers prescribed to children may detract from their all‐things‐considered health, as the available evidence for their effectiveness is weak or conflicting and we are learning more about the risks involved Biggs, M. (2023). The Dutch protocol for juvenile transsexuals: Origins and evidence. *Journal of Sex & Marital Therapy*, *49*(4), 348–368. It may turn out, however, that they do improve the all‐things‐considered health of children experiencing gender dysphoria.

29 Brummett, op. cit. note 6.

30 Giubilini, A. (2020). Conscientious objection in healthcare: Neither a negative nor a positive right. *The Journal of Clinical Ethics*, *31*(2), 146–153.

31 Ben‐Moshe, N. (2019). The internal morality of medicine: A constructivist approach. *Synthese, 196*(11), 4449–4467.

